# Intracranial extension of basal cell carcinoma despite negative radiographic evidence of direct bony neoplastic invasion

**DOI:** 10.1016/j.jdcr.2024.09.004

**Published:** 2024-09-24

**Authors:** Bahar Momin, Brooke A. Burgess, Gregory I. Kelts, Ryan P. Morton, Joshua L. Owen

**Affiliations:** a Long School of Medicine, University of Texas Health Science Center San Antonio, San Antonio, Texas; b Division of Dermatology, University of Texas Health Science Center San Antonio, San Antonio, Texas; c Division of Otolaryngology, Brooke Army Medical Center, Ft. Sam Houston, Texas; d Division of Neurosurgery, Brooke Army Medical Center, Ft. Sam Houston, Texas; e Dermatology Service, South Texas Veterans Health Care System, San Antonio, Texas

**Keywords:** basal cell carcinoma, diploic vein, dura, emissary vein, imaging, intracranial, Mohs micrographic surgery, skull

## Introduction

Basal cell carcinoma (BCC) is the most common cutaneous malignancy and, despite a typically indolent clinical course, can invade local structures. Intracranial BCC is a rare phenomenon that can occur secondary to distant metastasis, or more frequently via direct extension through calvarium. Radiographic imaging modalities such as computed tomography (CT), magnetic resonance imaging (MRI), and/or positron emission tomography (PET) are used to assess degree of bony invasion of the skull, which informs subsequent treatment planning. We present the case of a multiply recurrent postauricular scalp BCC that despite no evidence of local bony destruction on PET/CT and MRI, the tumor was subsequently found to invade parietal bone, dura mater, and sigmoid sinus.

## Case presentation

An 81-year-old male with a complex history of a large left postauricular scalp, neck, and ear BCC with multiple prior recurrences presented in 2023 with a 0.6 cm ulceration at the prior surgical site concerning for a third BCC recurrence.

**Fig 1:**
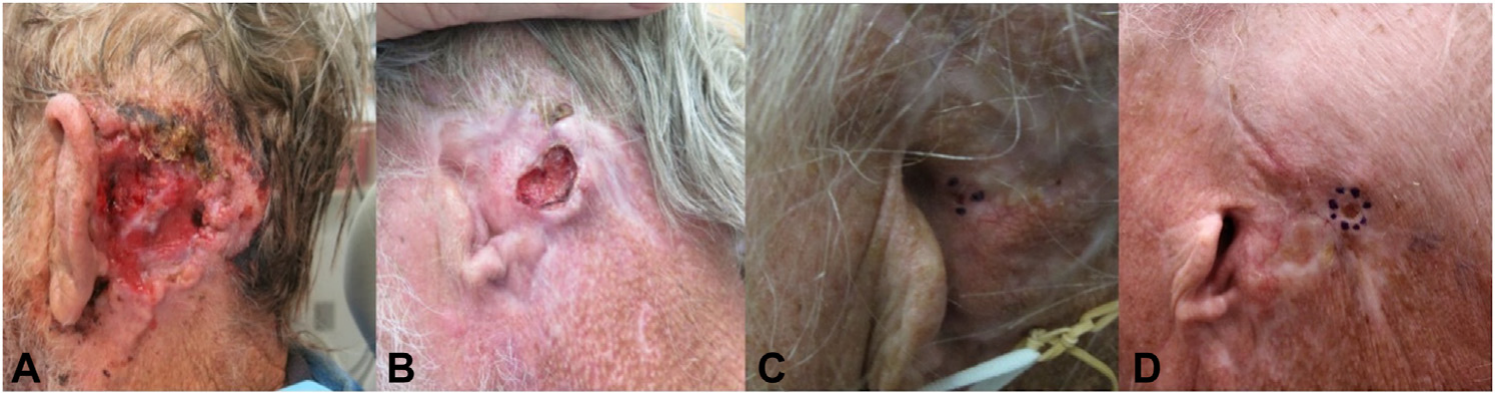
**A,** Patient on initial presentation with a large, fungating, bleeding plaque of the left postauricular scalp. **B,** First BCC recurrence with new, bleeding, ulcerated plaque. **C,** Second BCC recurrence with small, ulcerated papule. **D,** Third BCC recurrence with ulcerated papule of the postauricular scalp. *BCC*, Basal cell carcinoma.

The patient initially presented (2014) with a years-long history of a large, bleeding plaque of left postauricular scalp (Fig 1, *A*); pathology demonstrated nodular BCC. Patient underwent wide local excision and left partial auriculectomy by otolaryngology and was advised to undergo adjuvant radiation; however, it was not completed. Surgical site appeared well-healed until early 2017, at which time the patient presented with a new, bleeding ulceration at the prior surgical site which was histopathologically confirmed to be recurrent BCC (Fig 1, *B*). The patient underwent a 12-month course of vismodegib which achieved clinical clearance of the tumor. Five post-treatment scouting biopsies displayed no evidence of malignancy. Patient maintained close follow-up without clinical evidence of recurrence until 2020 when a second BCC recurrence of the left postauricular scalp was identified (Fig 1, *C*). The patient underwent 9 stages of Mohs micrographic surgery (MMS), achieved histologic clearance, and then completed adjuvant radiotherapy. One year after MMS (7 months after final radiation treatment), MRI did not show evidence of a residual mass or abnormal enhancement.

**Fig 2:**
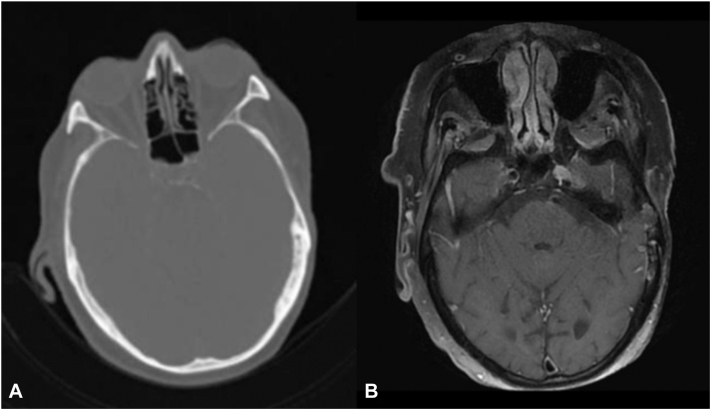
**A,** PET/CT demonstrating no evidence of local or bony invasion. Focal avidity at the left EAC (standardized uptake value 3.6). **B,** MRI head and neck: No abnormal calvarial bone mineralization, no soft tissue mass or mass-like enhancement, stable minimal soft tissue thickening within the left EAC, suspected to be consistent with chronic inflammatory change. *CT*, Computed tomography; *EAC*, external auditory canal; *MRI*, magnetic resonance imaging; *PET*, positron emission tomography.

Surgical site appeared well-healed until identification of recurrent BCC in 2023 (Fig 1, *D*). Whole body PET/CT was significant for new focal fluorodeoxyglucose (FDG) avidity (standardized uptake value 3.6) at the left external auditory canal (EAC) (Fig 2, *A*). Follow-up MRI was significant for no soft tissue mass, mass-like enhancement, or evidence of cortical erosion/bone marrow change (Fig 2, *B*). Stable minimal soft tissue thickening was identified within the EAC, and when correlated with the area of FDG avidity on PET/CT, was attributed to chronic inflammation secondary to mastoiditis.

**Fig 3:**
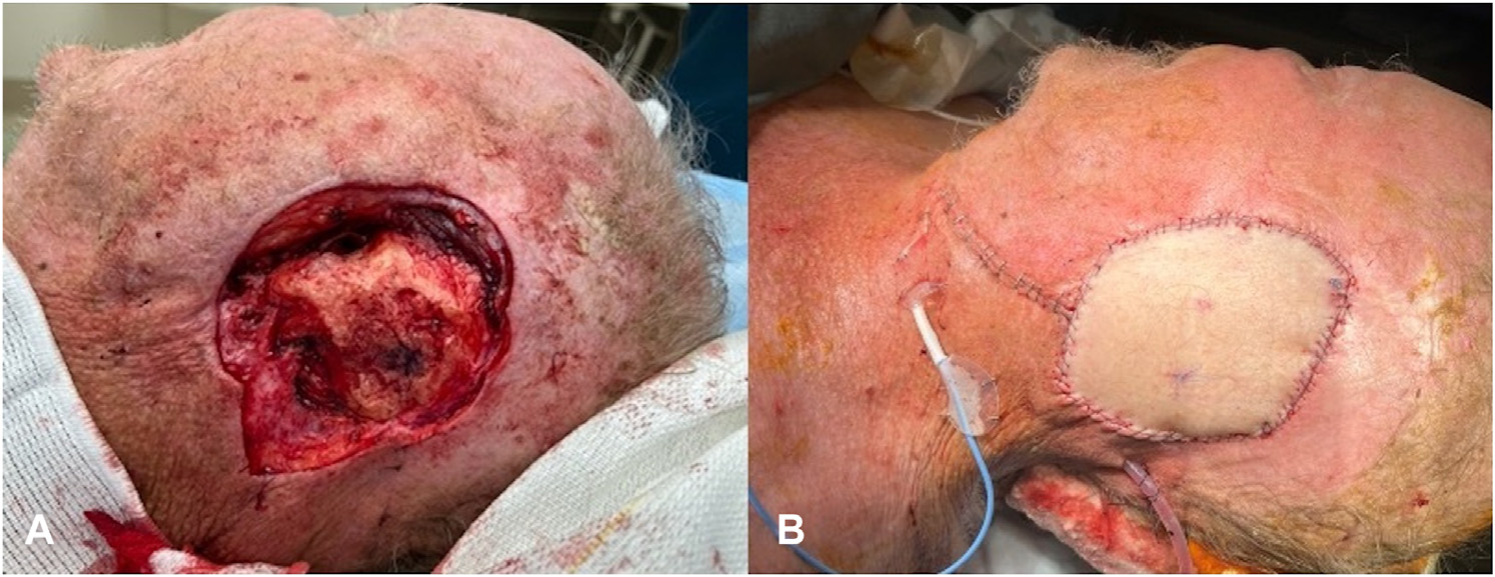
**A,** Defect after MMS in 2023 that displayed persistent BCC positivity of mastoid bone and EAC. Stippled temporal bone is visible, with a gentian violet-marked area of the bone demarcating location of initial stage. **B,** Postoperative appearance of anterolateral thigh free flap completed by otolaryngology after resection of temporal (including EAC) and parietal bones. *BCC*, basal cell carcinoma; *EAC*, external auditory canal; *MMS*, Mohs micrographic surgery.

**Fig 4:**
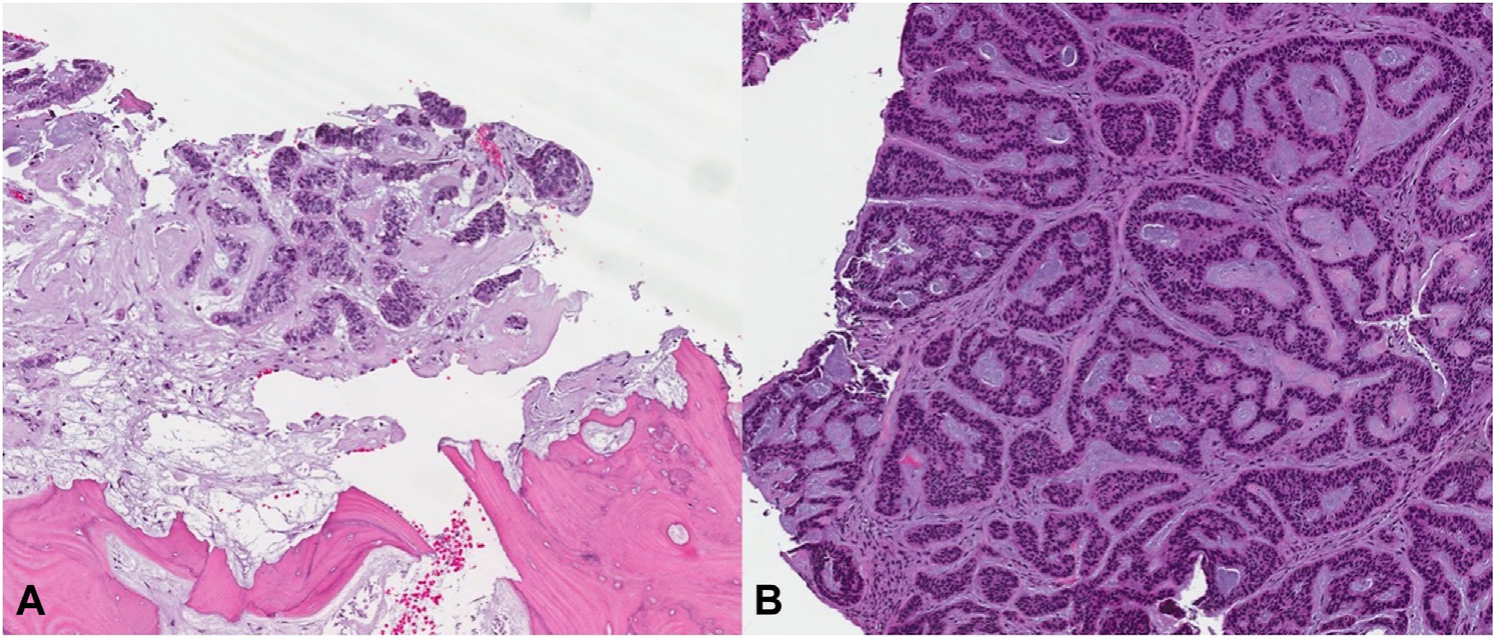
**A,** Surgical pathology of the parietal bone (hematoxylin and eosin, 10×) depicting fragments of parietal bone with involvement of BCC. **B,** Surgical pathology of dura mater (hematoxylin and eosin, 10×) with prominent infiltrative pattern of BCC. *BCC*, Basal cell carcinoma.

Multidisciplinary tumor board consensus was, given the lack of evidence of bony invasion on imaging, to proceed with MMS. After 5 stages, clear margins were achieved except for persistent tumor positivity of mastoid bone (deep margin) and bone and skin (peripheral and deep margins) within EAC (Fig 3, *A*). Given further resection was not possible under local anesthesia, MMS was halted. Patient underwent further excision by otolaryngology and neurosurgery under general anesthesia. Resection of temporal bone, EAC, and parietal bone revealed BCC involving parietal bone, underlying epidural space, mastoid contents, sigmoid sinus, and EAC (Fig 4). Due to BCC positivity of the dura mater and sigmoid sinus, complete resection was not possible. Intraoperative decision was made to forego further resection and treat with postoperative radiotherapy. Resultant defect was repaired via anterolateral thigh free flap by otolaryngology (Fig 3, *B*). Patient was discharged from the hospital on postoperative day 7, returned for scheduled outpatient follow-up, and was scheduled to begin radiotherapy 6 weeks after surgery. However, he suffered a myocardial infarction and died 5 weeks postoperatively.

## Discussion

Large, untreated BCCs of the face and scalp are high risk for local invasion and metastasis. The latency period between the development of BCC and subsequent metastasis or local bony invasion is about 9 years.^1^ Preoperative radiographic imaging can be helpful to assess tumor extent and depth. If gross bony invasion were present, imaging typically displays abnormal mineralization (CT), bone marrow changes (MRI), or abnormal FDG avidity (PET/CT).^2^ Imaging modalities have their limitations; for example, MRI has a higher false-positive rate for bony involvement compared to CT or PET/CT.^2^

It is therefore unusual for our patient to have BCC within parietal bone, underlying dura mater, and sigmoid sinus with both MRI and PET/CT without radiographic evidence of bony extension/invasion. We hypothesize that the underlying deeply persistent/recurrent BCC extended through the emissary veins which directly communicate between the scalp and the epidural space. The parietal bone, which was positive for tumor along with the temporal bone, has the highest density of both diploic vascular channels and emissary veins, both of which have capacity to facilitate direct invasion into the epidural space.^3^

There are no established treatment guidelines for intracranial BCC and intradural BCC is exceedingly rare and often results in death.^4^ Intracranial (and presumably intradural) BCC treatment typically entails aggressive tumor excision and subsequent reconstruction with or without neoadjuvant/adjuvant hedgehog pathway inhibitor, immunotherapy, or radiotherapy. Hedgehog pathway inhibitors like vismodegib and sonidegib represent potential treatment options for intradural BCC, and preclinical studies of sonidegib demonstrated penetration of blood-brain barrier.^5^ In addition, a case of cerebritis secondary to intracranial BCC resolved after 1 month of vismodegib, with complete tumor response on imaging after 6 months of vismodegib.^6^ In terms of radiotherapy for intradural BCC, Parizel et al^7^ report a case of intracerebral extension of BCC with tumor shrinkage after radiation therapy. Definitive treatment for our patient may have required a combination of medical and radiation treatment modalities.

This case highlights the complex behavior of long-standing, invasive BCC, and to the best of our knowledge, is the first documented case of intracranial BCC without positive radiographic evidence for bony neoplastic involvement. Clinicians should remain cognizant of possible diploic/emissary vein invasion and thus intracranial extension of BCC in the absence of gross cortical erosion on imaging.

## Conflicts of interest

None disclosed.
